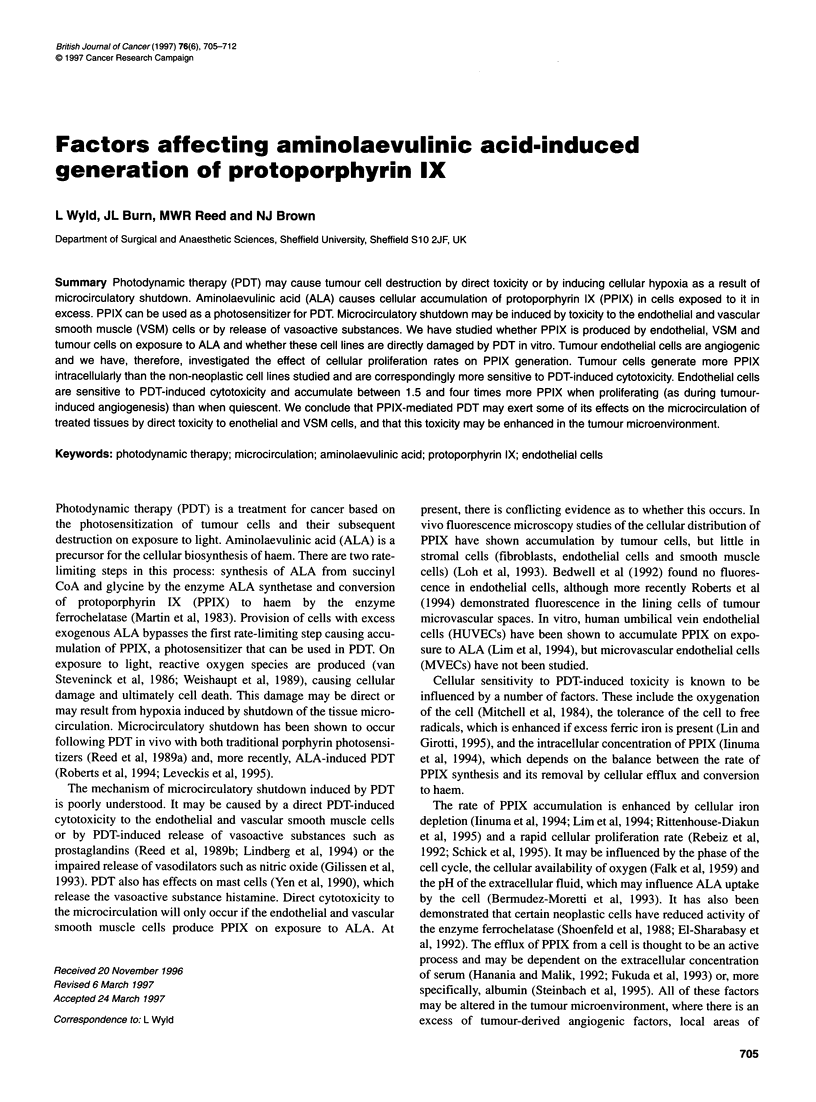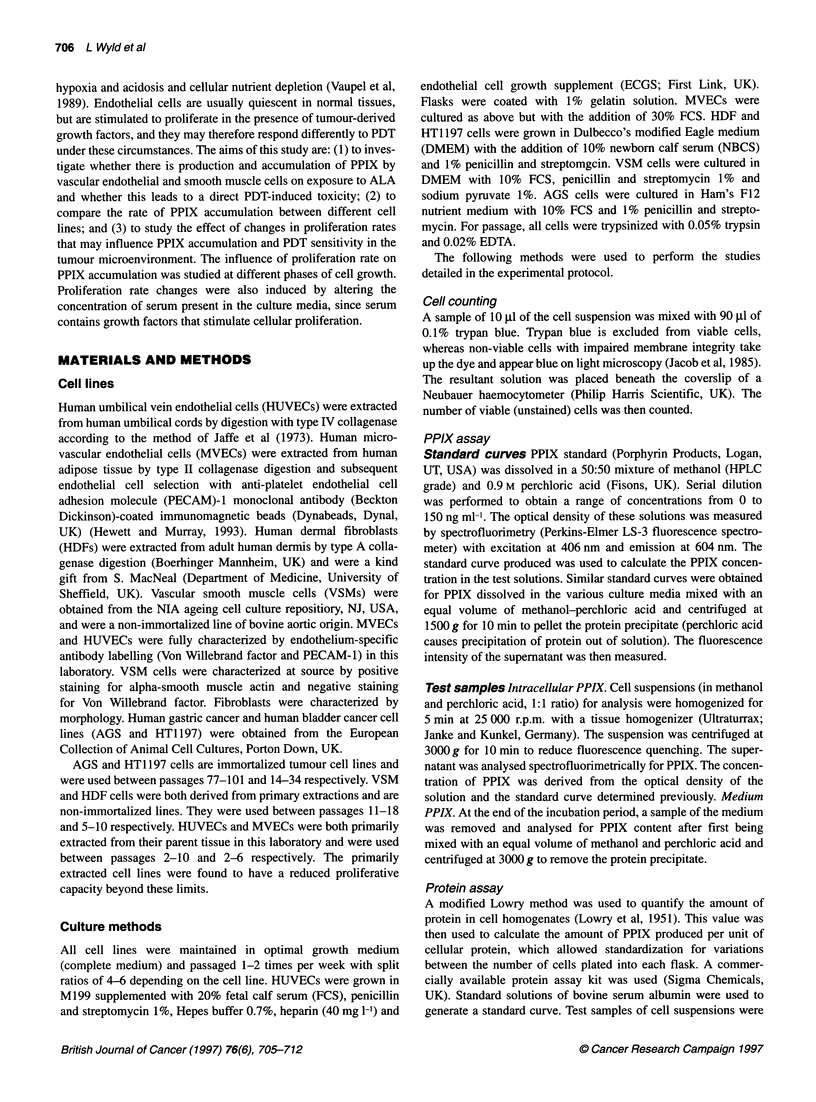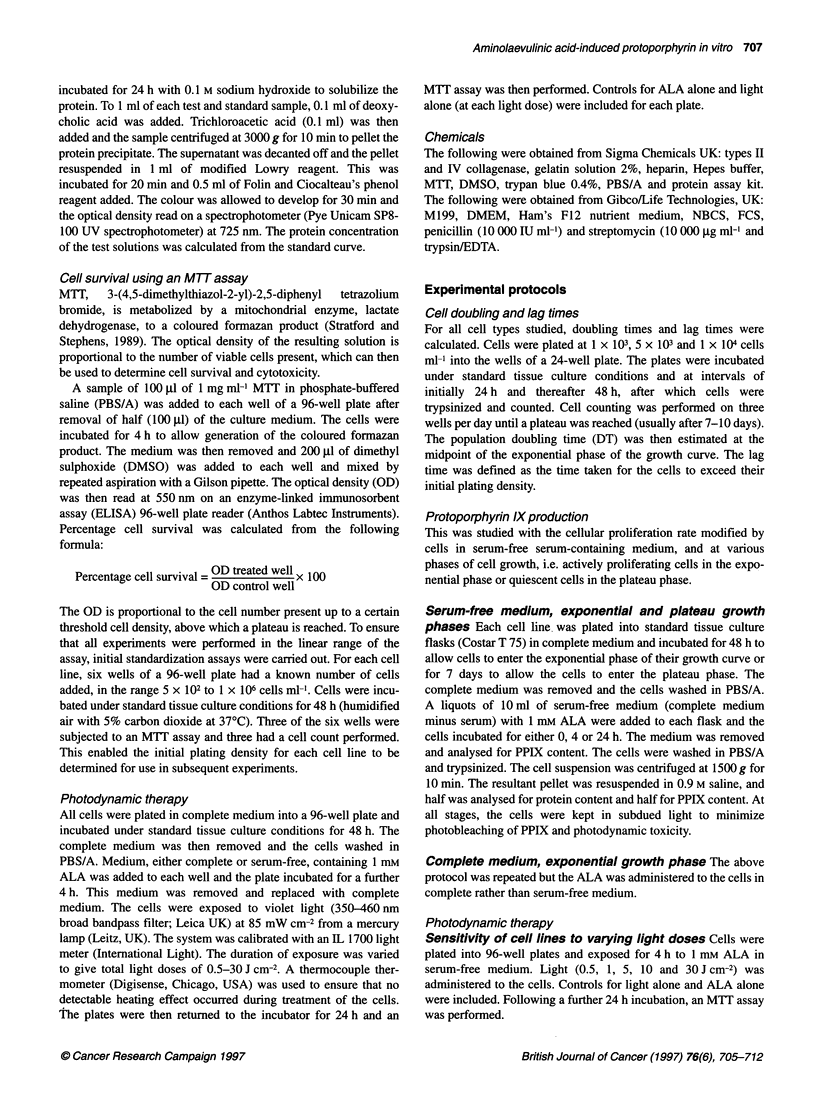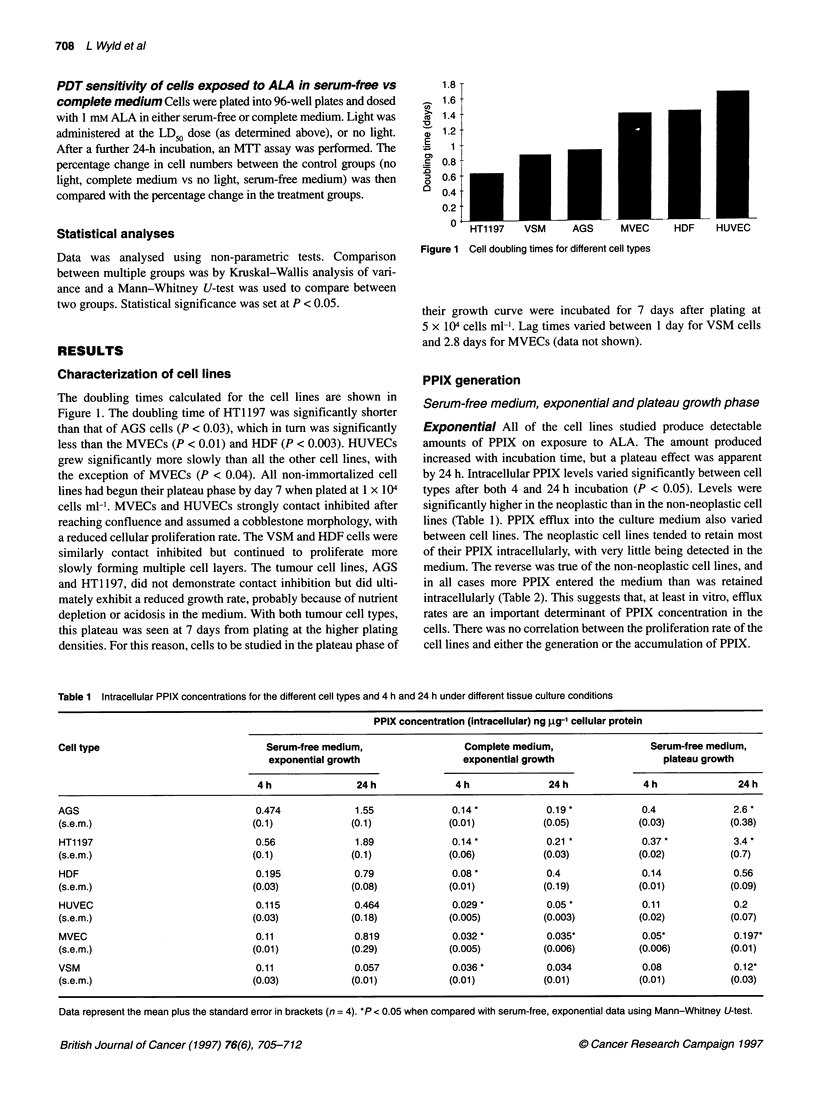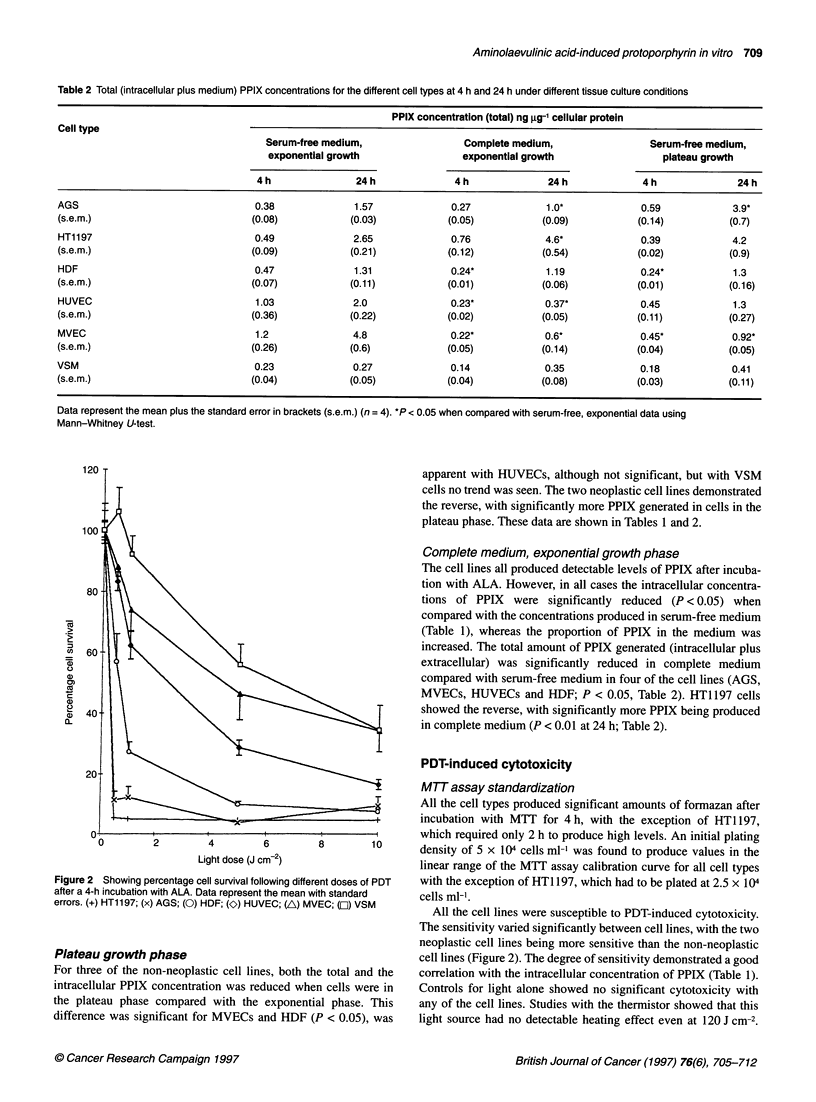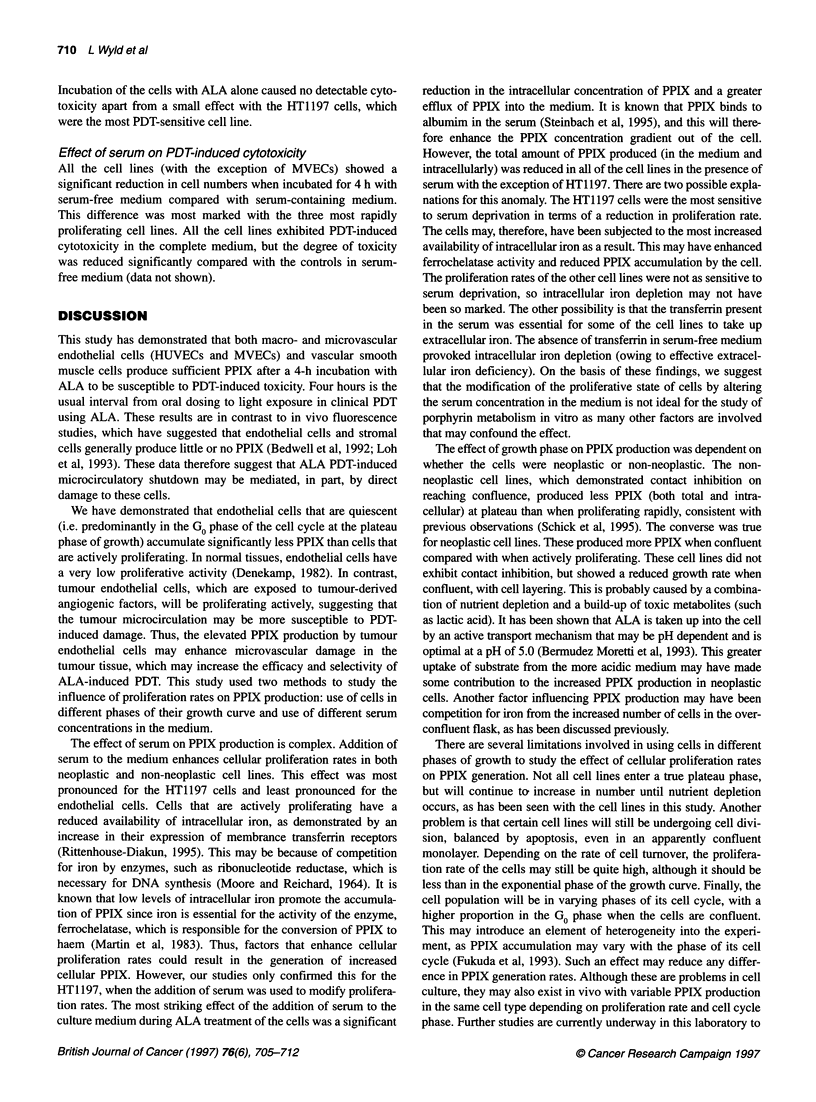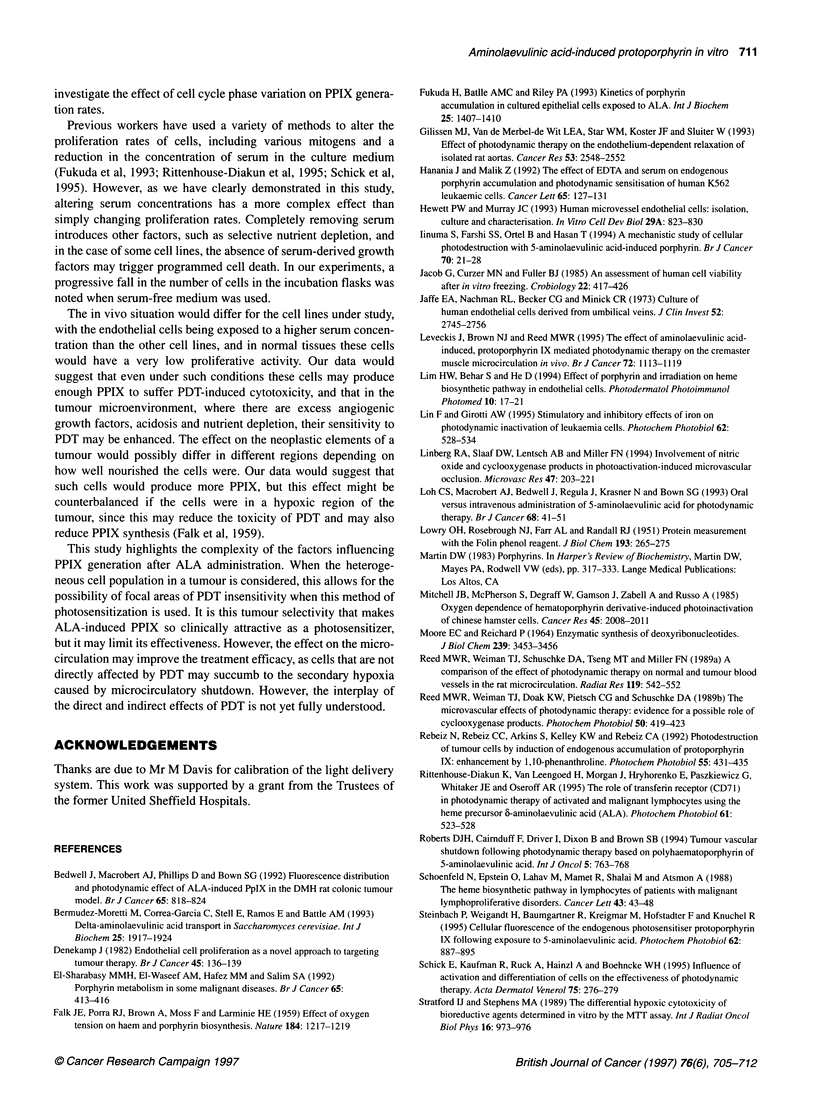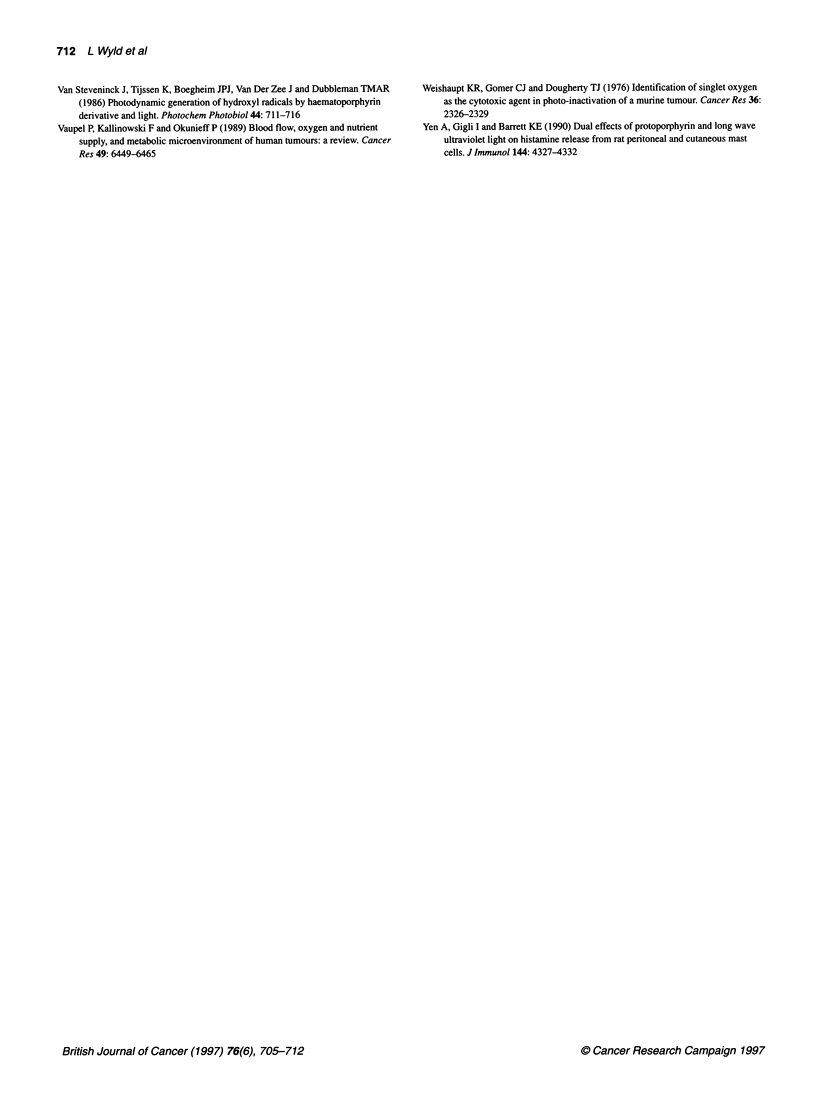# Factors affecting aminolaevulinic acid-induced generation of protoporphyrin IX.

**DOI:** 10.1038/bjc.1997.450

**Published:** 1997

**Authors:** L. Wyld, J. L. Burn, M. W. Reed, N. J. Brown

**Affiliations:** Department of Surgical and Anaesthetic Sciences, Sheffield University, UK.

## Abstract

Photodynamic therapy (PDT) may cause tumour cell destruction by direct toxicity or by inducing cellular hypoxia as a result of microcirculatory shutdown. Aminolaevulinic acid (ALA) causes cellular accumulation of protoporphyrin IX (PPIX) in cells exposed to it in excess. PPIX can be used as a photosensitizer for PDT. Microcirculatory shutdown may be induced by toxicity to the endothelial and vascular smooth muscle (VSM) cells or by release of vasoactive substances. We have studied whether PPIX is produced by endothelial, VSM and tumour cells on exposure to ALA and whether these cell lines are directly damaged by PDT in vitro. Tumour endothelial cells are angiogenic and we have, therefore, investigated the effect of cellular proliferation rates on PPIX generation. Tumour cells generate more PPIX intracellularly than the non-neoplastic cell lines studied and are correspondingly more sensitive to PDT-induced cytotoxicity. Endothelial cells are sensitive to PDT-induced cytotoxicity and accumulate between 1.5 and four times more PPIX when proliferating (as during tumour-induced angiogenesis) than when quiescent. We conclude that PPIX-mediated PDT may exert some of its effects on the microcirculation of treated tissues by direct toxicity to endothelial and VSM cells, and that this toxicity may be enhanced in the tumour microenvironment.


British Journal of Cancer (1997) 76(6), 705–712
© 1997 Cancer Research Campaign

### Factors affecting aminolaevulinic acid-induced generation of protoporphyrin IX

**L Wyld, JL Burn, MWR Reed and NJ Brown**

Department of Surgical and Anaesthetic Sciences, Sheffield University, Sheffield S10 2JF, UK

**Summary** Photodynamic therapy (PDT) may cause tumour cell destruction by direct toxicity or by inducing cellular hypoxia as a result of microcirculatory shutdown. Aminolaevulinic acid (ALA) causes cellular accumulation of protoporphyrin IX (PPIX) in cells exposed to it in excess. PPIX can be used as a photosensitizer for PDT. Microcirculatory shutdown may be induced by toxicity to the endothelial and vascular smooth muscle (VSM) cells or by release of vasoactive substances. We have studied whether PPIX is produced by endothelial, VSM and tumour cells on exposure to ALA and whether these cell lines are directly damaged by PDT in vitro. Tumour endothelial cells are angiogenic and we have, therefore, investigated the effect of cellular proliferation rates on PPIX generation. Tumour cells generate more PPIX intracellularly than the non-neoplastic cell lines studied and are correspondingly more sensitive to PDT-induced cytotoxicity. Endothelial cells are sensitive to PDT-induced cytotoxicity and accumulate between 1.5 and four times more PPIX when proliferating (as during tumour-induced angiogenesis) than when quiescent. We conclude that PPIX-mediated PDT may exert some of its effects on the microcirculation of treated tissues by direct toxicity to enothelial and VSM cells, and that this toxicity may be enhanced in the tumour microenvironment.

**Keywords:** photodynamic therapy; microcirculation; aminolaevulinic acid; protoporphyrin IX; endothelial cells

Photodynamic therapy (PDT) is a treatment for cancer based on the photosensitization of tumour cells and their subsequent destruction on exposure to light. Aminolaevulinic acid (ALA) is a precursor for the cellular biosynthesis of haem. There are two rate-limiting steps in this process: synthesis of ALA from succinyl CoA and glycine by the enzyme ALA synthetase and conversion of protoporphyrin IX (PPIX) to haem by the enzyme ferrochelatase (Martin et al, 1983). Provision of cells with excess exogenous ALA bypasses the first rate-limiting step causing accumulation of PPIX, a photosensitizer that can be used in PDT. On exposure to light, reactive oxygen species are produced (van Steveninck et al, 1986; Weishaupt et al, 1989), causing cellular damage and ultimately cell death. This damage may be direct or may result from hypoxia induced by shutdown of the tissue microcirculation. Microcirculatory shutdown has been shown to occur following PDT in vivo with both traditional porphyrin photosensitizers (Reed et al, 1989a) and, more recently, ALA-induced PDT (Roberts et al, 1994; Leveckis et al, 1995).

The mechanism of microcirculatory shutdown induced by PDT is poorly understood. It may be caused by a direct PDT-induced cytotoxicity to the endothelial and vascular smooth muscle cells or by PDT-induced release of vasoactive substances such as prostaglandins (Reed et al, 1989b; Lindberg et al, 1994) or the impaired release of vasodilators such as nitric oxide (Gilissen et al, 1993). PDT also has effects on mast cells (Yen et al, 1990), which release the vasoactive substance histamine. Direct cytotoxicity to the microcirculation will only occur if the endothelial and vascular smooth muscle cells produce PPIX on exposure to ALA. At present, there is conflicting evidence as to whether this occurs. In vivo fluorescence microscopy studies of the cellular distribution of PPIX have shown accumulation by tumour cells, but little in stromal cells (fibroblasts, endothelial cells and smooth muscle cells) (Loh et al, 1993). Bedwell et al (1992) found no fluorescence in endothelial cells, although more recently Roberts et al (1994) demonstrated fluorescence in the lining cells of tumour microvascular spaces. In vitro, human umbilical vein endothelial cells (HUVECs) have been shown to accumulate PPIX on exposure to ALA (Lim et al, 1994), but microvascular endothelial cells (MVECs) have not been studied.

*Received 20 November 1996*
*Revised 6 March 1997*
*Accepted 24 March 1997*

*Correspondence to:* L Wyld

Cellular sensitivity to PDT-induced toxicity is known to be influenced by a number of factors. These include the oxygenation of the cell (Mitchell et al, 1984), the tolerance of the cell to free radicals, which is enhanced if excess ferric iron is present (Lin and Girotti, 1995), and the intracellular concentration of PPIX (Iinuma et al, 1994), which depends on the balance between the rate of PPIX synthesis and its removal by cellular efflux and conversion to haem.

The rate of PPIX accumulation is enhanced by cellular iron depletion (Iinuma et al, 1994; Lim et al, 1994; Rittenhouse-Diakun et al, 1995) and a rapid cellular proliferation rate (Rebeiz et al, 1992; Schick et al, 1995). It may be influenced by the phase of the cell cycle, the cellular availability of oxygen (Falk et al, 1959) and the pH of the extracellular fluid, which may influence ALA uptake by the cell (Bermudez-Moretti et al, 1993). It has also been demonstrated that certain neoplastic cells have reduced activity of the enzyme ferrochelatase (Shoenfeld et al, 1988; El-Sharabasy et al, 1992). The efflux of PPIX from a cell is thought to be an active process and may be dependent on the extracellular concentration of serum (Hanania and Malik, 1992; Fukuda et al, 1993) or, more specifically, albumin (Steinbach et al, 1995). All of these factors may be altered in the tumour microenvironment, where there is an excess of tumour-derived angiogenic factors, local areas of hypoxia and acidosis and cellular nutrient depletion (Vaupel et al, 1989). Endothelial cells are usually quiescent in normal tissues, but are stimulated to proliferate in the presence of tumour-derived growth factors, and they may therefore respond differently to PDT under these circumstances. The aims of this study are: (1) to investigate whether there is production and accumulation of PPIX by vascular endothelial and smooth muscle cells on exposure to ALA and whether this leads to a direct PDT-induced toxicity; (2) to compare the rate of PPIX accumulation between different cell lines; and (3) to study the effect of changes in proliferation rates that may influence PPIX accumulation and PDT sensitivity in the tumour microenvironment. The influence of proliferation rate on PPIX accumulation was studied at different phases of cell growth. Proliferation rate changes were also induced by altering the concentration of serum present in the culture media, since serum contains growth factors that stimulate cellular proliferation.

### MATERIALS AND METHODS

### Cell lines

Human umbilical vein endothelial cells (HUVECs) were extracted from human umbilical cords by digestion with type IV collagenase according to the method of Jaffe et al (1973). Human microvascular endothelial cells (MVECs) were extracted from human adipose tissue by type II collagenase digestion and subsequent endothelial cell selection with anti-platelet endothelial cell adhesion molecule (PECAM)-1 monoclonal antibody (Beckton Dickinson)-coated immunomagnetic beads (Dynabeads, Dynal, UK) (Hewett and Murray, 1993). Human dermal fibroblasts (HDFs) were extracted from adult human dermis by type A collagenase digestion (Boerhinger Mannheim, UK) and were a kind gift from S. MacNeal (Department of Medicine, University of Sheffield, UK). Vascular smooth muscle cells (VSMs) were obtained from the NIA ageing cell culture repositiory, NJ, USA, and were a non-immortalized line of bovine aortic origin. MVECs and HUVECs were fully characterized by endothelium-specific antibody labelling (Von Willebrand factor and PECAM-1) in this laboratory. VSM cells were characterized at source by positive staining for alpha-smooth muscle actin and negative staining for Von Willebrand factor. Fibroblasts were characterized by morphology. Human gastric cancer and human bladder cancer cell lines (AGS and HT1197) were obtained from the European Collection of Animal Cell Cultures, Porton Down, UK.

AGS and HT1197 cells are immortalized tumour cell lines and were used between passages 77–101 and 14–34 respectively. VSM and HDF cells were both derived from primary extractions and are non-immortalized lines. They were used between passages 11–18 and 5–10 respectively. HUVECs and MVECs were both primarily extracted from their parent tissue in this laboratory and were used between passages 2–10 and 2–6 respectively. The primarily extracted cell lines were found to have a reduced proliferative capacity beyond these limits.

### Culture methods

All cell lines were maintained in optimal growth medium (complete medium) and passaged 1–2 times per week with split ratios of 4–6 depending on the cell line. HUVECs were grown in M199 supplemented with 20% fetal calf serum (FCS), penicillin and streptomycin 1%, Hepes buffer 0.7%, heparin (40 mg $l^{-1}$) and endothelial cell growth supplement (ECGS; First Link, UK). Flasks were coated with 1% gelatin solution. MVECs were cultured as above but with the addition of 30% FCS. HDF and HT1197 cells were grown in Dulbecco's modified Eagle medium (DMEM) with the addition of 10% newborn calf serum (NBCS) and 1% penicillin and streptomgcin. VSM cells were cultured in DMEM with 10% FCS, penicillin and streptomycin 1% and sodium pyruvate 1%. AGS cells were cultured in Ham's F12 nutrient medium with 10% FCS and 1% penicillin and streptomycin. For passage, all cells were trypsinized with 0.05% trypsin and 0.02% EDTA.

The following methods were used to perform the studies detailed in the experimental protocol.

#### *Cell counting*

A sample of 10 µl of the cell suspension was mixed with 90 µl of 0.1% trypan blue. Trypan blue is excluded from viable cells, whereas non-viable cells with impaired membrane integrity take up the dye and appear blue on light microscopy (Jacob et al, 1985). The resultant solution was placed beneath the coverslip of a Neubauer haemocytometer (Philip Harris Scientific, UK). The number of viable (unstained) cells was then counted.

#### *PPIX assay*

***Standard curves*** PPIX standard (Porphyrin Products, Logan, UT, USA) was dissolved in a 50:50 mixture of methanol (HPLC grade) and 0.9 M perchloric acid (Fisons, UK). Serial dilution was performed to obtain a range of concentrations from 0 to 150 ng $ml^{-1}$. The optical density of these solutions was measured by spectrofluorimetry (Perkins-Elmer LS-3 fluorescence spectrometer) with excitation at 406 nm and emission at 604 nm. The standard curve produced was used to calculate the PPIX concentration in the test solutions. Similar standard curves were obtained for PPIX dissolved in the various culture media mixed with an equal volume of methanol–perchloric acid and centrifuged at 1500 ***g*** for 10 min to pellet the protein precipitate (perchloric acid causes precipitation of protein out of solution). The fluorescence intensity of the supernatant was then measured.

***Test samples*** *Intracellular PPIX.* Cell suspensions (in methanol and perchloric acid, 1:1 ratio) for analysis were homogenized for 5 min at 25 000 r.p.m. with a tissue homogenizer (Ultraturrax; Janke and Kunkel, Germany). The suspension was centrifuged at 3000 ***g*** for 10 min to reduce fluorescence quenching. The supernatant was analysed spectrofluorimetrically for PPIX. The concentration of PPIX was derived from the optical density of the solution and the standard curve determined previously. *Medium PPIX.* At the end of the incubation period, a sample of the medium was removed and analysed for PPIX content after first being mixed with an equal volume of methanol and perchloric acid and centrifuged at 3000 ***g*** to remove the protein precipitate.

#### *Protein assay*

A modified Lowry method was used to quantify the amount of protein in cell homogenates (Lowry et al, 1951). This value was then used to calculate the amount of PPIX produced per unit of cellular protein, which allowed standardization for variations between the number of cells plated into each flask. A commercially available protein assay kit was used (Sigma Chemicals, UK). Standard solutions of bovine serum albumin were used to generate a standard curve. Test samples of cell suspensions were

 © Cancer Research Campaign 1997

incubated for 24 h with 0.1 M sodium hydroxide to solubilize the protein. To 1 ml of each test and standard sample, 0.1 ml of deoxycholic acid was added. Trichloroacetic acid (0.1 ml) was then added and the sample centrifuged at 3000 *g* for 10 min to pellet the protein precipitate. The supernatant was decanted off and the pellet resuspended in 1 ml of modified Lowry reagent. This was incubated for 20 min and 0.5 ml of Folin and Ciocalteau's phenol reagent added. The colour was allowed to develop for 30 min and the optical density read on a spectrophotometer (Pye Unicam SP8-100 UV spectrophotometer) at 725 nm. The protein concentration of the test solutions was calculated from the standard curve.

### *Cell survival using an MTT assay*

MTT, 3-(4,5-dimethylthiazol-2-yl)-2,5-diphenyl tetrazolium bromide, is metabolized by a mitochondrial enzyme, lactate dehydrogenase, to a coloured formazan product (Stratford and Stephens, 1989). The optical density of the resulting solution is proportional to the number of viable cells present, which can then be used to determine cell survival and cytotoxicity.

A sample of 100 μl of 1 mg $ml^{-1}$ MTT in phosphate-buffered saline (PBS/A) was added to each well of a 96-well plate after removal of half (100 μl) of the culture medium. The cells were incubated for 4 h to allow generation of the coloured formazan product. The medium was then removed and 200 μl of dimethyl sulphoxide (DMSO) was added to each well and mixed by repeated aspiration with a Gilson pipette. The optical density (OD) was then read at 550 nm on an enzyme-linked immunosorbent assay (ELISA) 96-well plate reader (Anthos Labtec Instruments). Percentage cell survival was calculated from the following formula:

$$\text{Percentage cell survival} = \frac{\text{OD treated well}}{\text{OD control well}} \times 100$$

The OD is proportional to the cell number present up to a certain threshold cell density, above which a plateau is reached. To ensure that all experiments were performed in the linear range of the assay, initial standardization assays were carried out. For each cell line, six wells of a 96-well plate had a known number of cells added, in the range $5 \times 10^2$ to $1 \times 10^6$ cells $ml^{-1}$. Cells were incubated under standard tissue culture conditions for 48 h (humidified air with 5% carbon dioxide at 37°C). Three of the six wells were subjected to an MTT assay and three had a cell count performed. This enabled the initial plating density for each cell line to be determined for use in subsequent experiments.

### *Photodynamic therapy*

All cells were plated in complete medium into a 96-well plate and incubated under standard tissue culture conditions for 48 h. The complete medium was then removed and the cells washed in PBS/A. Medium, either complete or serum-free, containing 1 mM ALA was added to each well and the plate incubated for a further 4 h. This medium was removed and replaced with complete medium. The cells were exposed to violet light (350–460 nm broad bandpass filter; Leica UK) at 85 mW $cm^{-2}$ from a mercury lamp (Leitz, UK). The system was calibrated with an IL 1700 light meter (International Light). The duration of exposure was varied to give total light doses of 0.5–30 J $cm^{-2}$. A thermocouple thermometer (Digisense, Chicago, USA) was used to ensure that no detectable heating effect occurred during treatment of the cells. The plates were then returned to the incubator for 24 h and an MTT assay was then performed. Controls for ALA alone and light alone (at each light dose) were included for each plate.

### *Chemicals*

The following were obtained from Sigma Chemicals UK: types II and IV collagenase, gelatin solution 2%, heparin, Hepes buffer, MTT, DMSO, trypan blue 0.4%, PBS/A and protein assay kit. The following were obtained from Gibco/Life Technologies, UK: M199, DMEM, Ham's F12 nutrient medium, NBCS, FCS, penicillin (10 000 IU $ml^{-1}$) and streptomycin (10 000 μg $ml^{-1}$ and trypsin/EDTA.

### Experimental protocols

### *Cell doubling and lag times*

For all cell types studied, doubling times and lag times were calculated. Cells were plated at $1 \times 10^3$, $5 \times 10^3$ and $1 \times 10^4$ cells $ml^{-1}$ into the wells of a 24-well plate. The plates were incubated under standard tissue culture conditions and at intervals of initially 24 h and thereafter 48 h, after which cells were trypsinized and counted. Cell counting was performed on three wells per day until a plateau was reached (usually after 7–10 days). The population doubling time (DT) was then estimated at the midpoint of the exponential phase of the growth curve. The lag time was defined as the time taken for the cells to exceed their initial plating density.

### *Protoporphyrin IX production*

This was studied with the cellular proliferation rate modified by cells in serum-free serum-containing medium, and at various phases of cell growth, i.e. actively proliferating cells in the exponential phase or quiescent cells in the plateau phase.

***Serum-free medium, exponential and plateau growth phases*** Each cell line was plated into standard tissue culture flasks (Costar T 75) in complete medium and incubated for 48 h to allow cells to enter the exponential phase of their growth curve or for 7 days to allow the cells to enter the plateau phase. The complete medium was removed and the cells washed in PBS/A. A liquots of 10 ml of serum-free medium (complete medium minus serum) with 1 mM ALA were added to each flask and the cells incubated for either 0, 4 or 24 h. The medium was removed and analysed for PPIX content. The cells were washed in PBS/A and trypsinized. The cell suspension was centrifuged at 1500 *g* for 10 min. The resultant pellet was resuspended in 0.9 M saline, and half was analysed for protein content and half for PPIX content. At all stages, the cells were kept in subdued light to minimize photobleaching of PPIX and photodynamic toxicity.

***Complete medium, exponential growth phase*** The above protocol was repeated but the ALA was administered to the cells in complete rather than serum-free medium.

### *Photodynamic therapy*

***Sensitivity of cell lines to varying light doses*** Cells were plated into 96-well plates and exposed for 4 h to 1 mM ALA in serum-free medium. Light (0.5, 1, 5, 10 and 30 J $cm^{-2}$) was administered to the cells. Controls for light alone and ALA alone were included. Following a further 24 h incubation, an MTT assay was performed.

© Cancer Research Campaign 1997

***PDT sensitivity of cells exposed to ALA in serum-free vs complete medium*** Cells were plated into 96-well plates and dosed with 1 mM ALA in either serum-free or complete medium. Light was administered at the $LD_{50}$ dose (as determined above), or no light. After a further 24-h incubation, an MTT assay was performed. The percentage change in cell numbers between the control groups (no light, complete medium vs no light, serum-free medium) was then compared with the percentage change in the treatment groups.

### Statistical analyses

Data was analysed using non-parametric tests. Comparison between multiple groups was by Kruskal–Wallis analysis of variance and a Mann–Whitney *U*-test was used to compare between two groups. Statistical significance was set at $P < 0.05$.

### RESULTS

### Characterization of cell lines

The doubling times calculated for the cell lines are shown in Figure 1. The doubling time of HT1197 was significantly shorter than that of AGS cells ($P < 0.03$), which in turn was significantly less than the MVECs ($P < 0.01$) and HDF ($P < 0.003$). HUVECs grew significantly more slowly than all the other cell lines, with the exception of MVECs ($P < 0.04$). All non-immortalized cell lines had begun their plateau phase by day 7 when plated at $1 \times 10^4$ cells $ml^{-1}$. MVECs and HUVECs strongly contact inhibited after reaching confluence and assumed a cobblestone morphology, with a reduced cellular proliferation rate. The VSM and HDF cells were similarly contact inhibited but continued to proliferate more slowly forming multiple cell layers. The tumour cell lines, AGS and HT1197, did not demonstrate contact inhibition but did ultimately exhibit a reduced growth rate, probably because of nutrient depletion or acidosis in the medium. With both tumour cell types, this plateau was seen at 7 days from plating at the higher plating densities. For this reason, cells to be studied in the plateau phase of their growth curve were incubated for 7 days after plating at $5 \times 10^4$ cells $ml^{-1}$. Lag times varied between 1 day for VSM cells and 2.8 days for MVECs (data not shown).

**Figure 1** Cell doubling times for different cell types

### PPIX generation

*Serum-free medium, exponential and plateau growth phase*

***Exponential*** All of the cell lines studied produce detectable amounts of PPIX on exposure to ALA. The amount produced increased with incubation time, but a plateau effect was apparent by 24 h. Intracellular PPIX levels varied significantly between cell types after both 4 and 24 h incubation ($P < 0.05$). Levels were significantly higher in the neoplastic than in the non-neoplastic cell lines (Table 1). PPIX efflux into the culture medium also varied between cell lines. The neoplastic cell lines tended to retain most of their PPIX intracellularly, with very little being detected in the medium. The reverse was true of the non-neoplastic cell lines, and in all cases more PPIX entered the medium than was retained intracellularly (Table 2). This suggests that, at least in vitro, efflux rates are an important determinant of PPIX concentration in the cells. There was no correlation between the proliferation rate of the cell lines and either the generation or the accumulation of PPIX.

**Table 1** Intracellular PPIX concentrations for the different cell types and 4 h and 24 h under different tissue culture conditions

| Cell type | PPIX concentration (intracellular) ng μg⁻¹ cellular protein | | | | | |
|---|---|---|---|---|---|---|
| | Serum-free medium, exponential growth | | Complete medium, exponential growth | | Serum-free medium, plateau growth | |
| | 4 h | 24 h | 4 h | 24 h | 4 h | 24 h |
| AGS | 0.474 | 1.55 | 0.14 * | 0.19 * | 0.4 | 2.6 * |
| (s.e.m.) | (0.1) | (0.1) | (0.01) | (0.05) | (0.03) | (0.38) |
| HT1197 | 0.56 | 1.89 | 0.14 * | 0.21 * | 0.37 * | 3.4 * |
| (s.e.m.) | (0.1) | (0.1) | (0.06) | (0.03) | (0.02) | (0.7) |
| HDF | 0.195 | 0.79 | 0.08 * | 0.4 | 0.14 | 0.56 |
| (s.e.m.) | (0.03) | (0.08) | (0.01) | (0.19) | (0.01) | (0.09) |
| HUVEC | 0.115 | 0.464 | 0.029 * | 0.05 * | 0.11 | 0.2 |
| (s.e.m.) | (0.03) | (0.18) | (0.005) | (0.003) | (0.02) | (0.07) |
| MVEC | 0.11 | 0.819 | 0.032 * | 0.035* | 0.05* | 0.197* |
| (s.e.m.) | (0.01) | (0.29) | (0.005) | (0.006) | (0.006) | (0.01) |
| VSM | 0.11 | 0.057 | 0.036 * | 0.034 | 0.08 | 0.12* |
| (s.e.m.) | (0.03) | (0.01) | (0.01) | (0.01) | (0.01) | (0.03) |

Data represent the mean plus the standard error in brackets ($n = 4$). *$P < 0.05$ when compared with serum-free, exponential data using Mann–Whitney *U*-test.

 © Cancer Research Campaign 1997

**Table 2** Total (intracellular plus medium) PPIX concentrations for the different cell types at 4 h and 24 h under different tissue culture conditions

| Cell type | PPIX concentration (total) ng $\mu g^{-1}$ cellular protein | | | | | |
|---|---|---|---|---|---|---|
| | Serum-free medium, exponential growth | | Complete medium, exponential growth | | Serum-free medium, plateau growth | |
| | 4 h | 24 h | 4 h | 24 h | 4 h | 24 h |
| AGS | 0.38 | 1.57 | 0.27 | 1.0* | 0.59 | 3.9* |
| (s.e.m.) | (0.08) | (0.03) | (0.05) | (0.09) | (0.14) | (0.7) |
| HT1197 | 0.49 | 2.65 | 0.76 | 4.6* | 0.39 | 4.2 |
| (s.e.m.) | (0.09) | (0.21) | (0.12) | (0.54) | (0.02) | (0.9) |
| HDF | 0.47 | 1.31 | 0.24* | 1.19 | 0.24* | 1.3 |
| (s.e.m.) | (0.07) | (0.11) | (0.01) | (0.06) | (0.01) | (0.16) |
| HUVEC | 1.03 | 2.0 | 0.23* | 0.37* | 0.45 | 1.3 |
| (s.e.m.) | (0.36) | (0.22) | (0.02) | (0.05) | (0.11) | (0.27) |
| MVEC | 1.2 | 4.8 | 0.22* | 0.6* | 0.45* | 0.92* |
| (s.e.m.) | (0.26) | (0.6) | (0.05) | (0.14) | (0.04) | (0.05) |
| VSM | 0.23 | 0.27 | 0.14 | 0.35 | 0.18 | 0.41 |
| (s.e.m.) | (0.04) | (0.05) | (0.04) | (0.08) | (0.03) | (0.11) |

Data represent the mean plus the standard error in brackets (s.e.m.) ($n = 4$). *$P < 0.05$ when compared with serum-free, exponential data using Mann–Whitney *U*-test.

**Figure 2** Showing percentage cell survival following different doses of PDT after a 4-h incubation with ALA. Data represent the mean with standard errors. (+) HT1197; (x) AGS; (O) HDF; (◇) HUVEC; (△) MVEC; (□) VSM

### *Plateau growth phase*

For three of the non-neoplastic cell lines, both the total and the intracellular PPIX concentration was reduced when cells were in the plateau phase compared with the exponential phase. This difference was significant for MVECs and HDF ($P < 0.05$), was apparent with HUVECs, although not significant, but with VSM cells no trend was seen. The two neoplastic cell lines demonstrated the reverse, with significantly more PPIX generated in cells in the plateau phase. These data are shown in Tables 1 and 2.

### *Complete medium, exponential growth phase*

The cell lines all produced detectable levels of PPIX after incubation with ALA. However, in all cases the intracellular concentrations of PPIX were significantly reduced ($P < 0.05$) when compared with the concentrations produced in serum-free medium (Table 1), whereas the proportion of PPIX in the medium was increased. The total amount of PPIX generated (intracellular plus extracellular) was significantly reduced in complete medium compared with serum-free medium in four of the cell lines (AGS, MVECs, HUVECs and HDF; $P < 0.05$, Table 2). HT1197 cells showed the reverse, with significantly more PPIX being produced in complete medium ($P < 0.01$ at 24 h; Table 2).

### PDT-induced cytotoxicity

### *MTT assay standardization*

All the cell types produced significant amounts of formazan after incubation with MTT for 4 h, with the exception of HT1197, which required only 2 h to produce high levels. An initial plating density of $5 \times 10^4$ cells ml$^{-1}$ was found to produce values in the linear range of the MTT assay calibration curve for all cell types with the exception of HT1197, which had to be plated at $2.5 \times 10^4$ cells ml$^{-1}$.

All the cell lines were susceptible to PDT-induced cytotoxicity. The sensitivity varied significantly between cell lines, with the two neoplastic cell lines being more sensitive than the non-neoplastic cell lines (Figure 2). The degree of sensitivity demonstrated a good correlation with the intracellular concentration of PPIX (Table 1). Controls for light alone showed no significant cytotoxicity with any of the cell lines. Studies with the thermistor showed that this light source had no detectable heating effect even at 120 J cm$^{-2}$.

© Cancer Research Campaign 1997

Incubation of the cells with ALA alone caused no detectable cytotoxicity apart from a small effect with the HT1197 cells, which were the most PDT-sensitive cell line.

### Effect of serum on PDT-induced cytotoxicity

All the cell lines (with the exception of MVECs) showed a significant reduction in cell numbers when incubated for 4 h with serum-free medium compared with serum-containing medium. This difference was most marked with the three most rapidly proliferating cell lines. All the cell lines exhibited PDT-induced cytotoxicity in the complete medium, but the degree of toxicity was reduced significantly compared with the controls in serum-free medium (data not shown).

### DISCUSSION

This study has demonstrated that both macro- and microvascular endothelial cells (HUVECs and MVECs) and vascular smooth muscle cells produce sufficient PPIX after a 4-h incubation with ALA to be susceptible to PDT-induced toxicity. Four hours is the usual interval from oral dosing to light exposure in clinical PDT using ALA. These results are in contrast to in vivo fluorescence studies, which have suggested that endothelial cells and stromal cells generally produce little or no PPIX (Bedwell et al, 1992; Loh et al, 1993). These data therefore suggest that ALA PDT-induced microcirculatory shutdown may be mediated, in part, by direct damage to these cells.

We have demonstrated that endothelial cells that are quiescent (i.e. predominantly in the $G_0$ phase of the cell cycle at the plateau phase of growth) accumulate significantly less PPIX than cells that are actively proliferating. In normal tissues, endothelial cells have a very low proliferative activity (Denekamp, 1982). In contrast, tumour endothelial cells, which are exposed to tumour-derived angiogenic factors, will be proliferating actively, suggesting that the tumour microcirculation may be more susceptible to PDT-induced damage. Thus, the elevated PPIX production by tumour endothelial cells may enhance microvascular damage in the tumour tissue, which may increase the efficacy and selectivity of ALA-induced PDT. This study used two methods to study the influence of proliferation rates on PPIX production: use of cells in different phases of their growth curve and use of different serum concentrations in the medium.

The effect of serum on PPIX production is complex. Addition of serum to the medium enhances cellular proliferation rates in both neoplastic and non-neoplastic cell lines. This effect was most pronounced for the HT1197 cells and least pronounced for the endothelial cells. Cells that are actively proliferating have a reduced availability of intracellular iron, as demonstrated by an increase in their expression of membrance transferrin receptors (Rittenhouse-Diakun, 1995). This may be because of competition for iron by enzymes, such as ribonucleotide reductase, which is necessary for DNA synthesis (Moore and Reichard, 1964). It is known that low levels of intracellular iron promote the accumulation of PPIX since iron is essential for the activity of the enzyme, ferrochelatase, which is responsible for the conversion of PPIX to haem (Martin et al, 1983). Thus, factors that enhance cellular proliferation rates could result in the generation of increased cellular PPIX. However, our studies only confirmed this for the HT1197, when the addition of serum was used to modify proliferation rates. The most striking effect of the addition of serum to the culture medium during ALA treatment of the cells was a significant reduction in the intracellular concentration of PPIX and a greater efflux of PPIX into the medium. It is known that PPIX binds to albumim in the serum (Steinbach et al, 1995), and this will therefore enhance the PPIX concentration gradient out of the cell. However, the total amount of PPIX produced (in the medium and intracellularly) was reduced in all of the cell lines in the presence of serum with the exception of HT1197. There are two possible explanations for this anomaly. The HT1197 cells were the most sensitive to serum deprivation in terms of a reduction in proliferation rate. The cells may, therefore, have been subjected to the most increased availability of intracellular iron as a result. This may have enhanced ferrochelatase activity and reduced PPIX accumulation by the cell. The proliferation rates of the other cell lines were not as sensitive to serum deprivation, so intracellular iron depletion may not have been so marked. The other possibility is that the transferrin present in the serum was essential for some of the cell lines to take up extracellular iron. The absence of transferrin in serum-free medium provoked intracellular iron depletion (owing to effective extracellular iron deficiency). On the basis of these findings, we suggest that the modification of the proliferative state of cells by altering the serum concentration in the medium is not ideal for the study of porphyrin metabolism in vitro as many other factors are involved that may confound the effect.

The effect of growth phase on PPIX production was dependent on whether the cells were neoplastic or non-neoplastic. The non-neoplastic cell lines, which demonstrated contact inhibition on reaching confluence, produced less PPIX (both total and intracellular) at plateau than when proliferating rapidly, consistent with previous observations (Schick et al, 1995). The converse was true for neoplastic cell lines. These produced more PPIX when confluent compared with when actively proliferating. These cell lines did not exhibit contact inhibition, but showed a reduced growth rate when confluent, with cell layering. This is probably caused by a combination of nutrient depletion and a build-up of toxic metabolites (such as lactic acid). It has been shown that ALA is taken up into the cell by an active transport mechanism that may be pH dependent and is optimal at a pH of 5.0 (Bermudez Moretti et al, 1993). This greater uptake of substrate from the more acidic medium may have made some contribution to the increased PPIX production in neoplastic cells. Another factor influencing PPIX production may have been competition for iron from the increased number of cells in the over-confluent flask, as has been discussed previously.

There are several limitations involved in using cells in different phases of growth to study the effect of cellular proliferation rates on PPIX generation. Not all cell lines enter a true plateau phase, but will continue to increase in number until nutrient depletion occurs, as has been seen with the cell lines in this study. Another problem is that certain cell lines will still be undergoing cell division, balanced by apoptosis, even in an apparently confluent monolayer. Depending on the rate of cell turnover, the proliferation rate of the cells may still be quite high, although it should be less than in the exponential phase of the growth curve. Finally, the cell population will be in varying phases of its cell cycle, with a higher proportion in the $G_0$ phase when the cells are confluent. This may introduce an element of heterogeneity into the experiment, as PPIX accumulation may vary with the phase of its cell cycle (Fukuda et al, 1993). Such an effect may reduce any difference in PPIX generation rates. Although these are problems in cell culture, they may also exist in vivo with variable PPIX production in the same cell type depending on proliferation rate and cell cycle phase. Further studies are currently underway in this laboratory to

© Cancer Research Campaign 1997

investigate the effect of cell cycle phase variation on PPIX generation rates.

Previous workers have used a variety of methods to alter the proliferation rates of cells, including various mitogens and a reduction in the concentration of serum in the culture medium (Fukuda et al, 1993; Rittenhouse-Diakun et al, 1995; Schick et al, 1995). However, as we have clearly demonstrated in this study, altering serum concentrations has a more complex effect than simply changing proliferation rates. Completely removing serum introduces other factors, such as selective nutrient depletion, and in the case of some cell lines, the absence of serum-derived growth factors may trigger programmed cell death. In our experiments, a progressive fall in the number of cells in the incubation flasks was noted when serum-free medium was used.

The in vivo situation would differ for the cell lines under study, with the endothelial cells being exposed to a higher serum concentration than the other cell lines, and in normal tissues these cells would have a very low proliferative activity. Our data would suggest that even under such conditions these cells may produce enough PPIX to suffer PDT-induced cytotoxicity, and that in the tumour microenvironment, where there are excess angiogenic growth factors, acidosis and nutrient depletion, their sensitivity to PDT may be enhanced. The effect on the neoplastic elements of a tumour would possibly differ in different regions depending on how well nourished the cells were. Our data would suggest that such cells would produce more PPIX, but this effect might be counterbalanced if the cells were in a hypoxic region of the tumour, since this may reduce the toxicity of PDT and may also reduce PPIX synthesis (Falk et al, 1959).

This study highlights the complexity of the factors influencing PPIX generation after ALA administration. When the heterogeneous cell population in a tumour is considered, this allows for the possibility of focal areas of PDT insensitivity when this method of photosensitization is used. It is this tumour selectivity that makes ALA-induced PPIX so clinically attractive as a photosensitizer, but it may limit its effectiveness. However, the effect on the microcirculation may improve the treatment efficacy, as cells that are not directly affected by PDT may succumb to the secondary hypoxia caused by microcirculatory shutdown. However, the interplay of the direct and indirect effects of PDT is not yet fully understood.

### ACKNOWLEDGEMENTS

Thanks are due to Mr M Davis for calibration of the light delivery system. This work was supported by a grant from the Trustees of the former United Sheffield Hospitals.

### REFERENCES

Bedwell J, Macrobert AJ, Phillips D and Bown SG (1992) Fluorescence distribution and photodynamic effect of ALA-induced PpIX in the DMH rat colonic tumour model. *Br J Cancer* **65**: 818–824

Bermudez-Moretti M, Correa-Garcia C, Stell E, Ramos E and Battle AM (1993) Delta-aminolaevulinic acid transport in *Saccharomyces cerevisiae*. *Int J Biochem* **25**: 1917–1924

Denekamp J (1982) Endothelial cell proliferation as a novel approach to targeting tumour therapy. *Br J Cancer* **45**: 136–139

El-Sharabasy MMH, El-Waseef AM, Hafez MM and Salim SA (1992) Porphyrin metabolism in some malignant diseases. *Br J Cancer* **65**: 413–416

Falk JE, Porra RJ, Brown A, Moss F and Larminie HE (1959) Effect of oxygen tension on haem and porphyrin biosynthesis. *Nature* **184**: 1217–1219

Fukuda H, Batlle AMC and Riley PA (1993) Kinetics of porphyrin accumulation in cultured epithelial cells exposed to ALA. *Int J Biochem* **25**: 1407–1410

Gilissen MJ, Van de Merbel-de Wit LEA, Star WM, Koster JF and Sluiter W (1993) Effect of photodynamic therapy on the endothelium-dependent relaxation of isolated rat aortas. *Cancer Res* **53**: 2548–2552

Hanania J and Malik Z (1992) The effect of EDTA and serum on endogenous porphyrin accumulation and photodynamic sensitisation of human K562 leukaemic cells. *Cancer Lett* **65**: 127–131

Hewett PW and Murray JC (1993) Human microvessel endothelial cells: isolation, culture and characterisation. *In Vitro Cell Dev Biol* **29A**: 823–830

Iinuma S, Farshi SS, Ortel B and Hasan T (1994) A mechanistic study of cellular photodestruction with 5-aminolaevulinic acid-induced porphyrin. *Br J Cancer* **70**: 21–28

Jacob G, Curzer MN and Fuller BJ (1985) An assessment of human cell viability after *in vitro* freezing. *Crobiology* **22**: 417–426

Jaffe EA, Nachman RL, Becker CG and Minick CR (1973) Culture of human endothelial cells derived from umbilical veins. *J Clin Invest* **52**: 2745–2756

Leveckis J, Brown NJ and Reed MWR (1995) The effect of aminolaevulinic acid-induced, protoporphyrin IX mediated photodynamic therapy on the cremaster muscle microcirculation *in vivo*. *Br J Cancer* **72**: 1113–1119

Lim HW, Behar S and He D (1994) Effect of porphyrin and irradiation on heme biosynthetic pathway in endothelial cells. *Photodermatol Photoimmunol Photomed* **10**: 17–21

Lin F and Girotti AW (1995) Stimulatory and inhibitory effects of iron on photodynamic inactivation of leukaemia cells. *Photochem Photobiol* **62**: 528–534

Linberg RA, Slaaf DW, Lentsch AB and Miller FN (1994) Involvement of nitric oxide and cyclooxygenase products in photoactivation-induced microvascular occlusion. *Microvasc Res* **47**: 203–221

Loh CS, Macrobert AJ, Bedwell J, Regula J, Krasner N and Bown SG (1993) Oral versus intravenous administration of 5-aminolaevulinic acid for photodynamic therapy. *Br J Cancer* **68**: 41–51

Lowry OH, Rosebrough NJ, Farr AL and Randall RJ (1951) Protein measurement with the Folin phenol reagent. *J Biol Chem* **193**: 265–275

Martin DW (1983) Porphyrins. In *Harper's Review of Biochemistry*, Martin DW, Mayes PA, Rodwell VW (eds), pp. 317–333. Lange Medical Publications: Los Altos, CA

Mitchell JB, McPherson S, Degraff W, Gamson J, Zabell A and Russo A (1985) Oxygen dependence of hematoporphyrin derivative-induced photoinactivation of chinese hamster cells. *Cancer Res* **45**: 2008–2011

Moore EC and Reichard P (1964) Enzymatic synthesis of deoxyribonucleotides. *J Biol Chem* **239**: 3453–3456

Reed MWR, Weiman TJ, Schuschke DA, Tseng MT and Miller FN (1989a) A comparison of the effect of photodynamic therapy on normal and tumour blood vessels in the rat microcirculation. *Radiat Res* **119**: 542–552

Reed MWR, Weiman TJ, Doak KW, Pietsch CG and Schuschke DA (1989b) The microvascular effects of photodynamic therapy: evidence for a possible role of cyclooxygenase products. *Photochem Photobiol* **50**: 419–423

Rebeiz N, Rebeiz CC, Arkins S, Kelley KW and Rebeiz CA (1992) Photodestruction of tumour cells by induction of endogenous accumulation of protoporphyrin IX: enhancement by 1,10-phenanthroline. *Photochem Photobiol* **55**: 431–435

Rittenhouse-Diakun K, Van Leengoed H, Morgan J, Hryhorenko E, Paszkiewicz G, Whitaker JE and Oseroff AR (1995) The role of transferin receptor (CD71) in photodynamic therapy of activated and malignant lymphocytes using the heme precursor δ-aminolaevulinic acid (ALA). *Photochem Photobiol* **61**: 523–528

Roberts DJH, Cairnduff F, Driver I, Dixon B and Brown SB (1994) Tumour vascular shutdown following photodynamic therapy based on polyhaematoporphyrin of 5-aminolaevulinic acid. *Int J Oncol* **5**: 763–768

Schoenfeld N, Epstein O, Lahav M, Mamet R, Shalai M and Atsmon A (1988) The heme biosynthetic pathway in lymphocytes of patients with malignant lymphoproliferative disorders. *Cancer Lett* **43**: 43–48

Steinbach P, Weigandt H, Baumgartner R, Kreigmar M, Hofstadter F and Knuchel R (1995) Cellular fluorescence of the endogenous photosensitiser protoporphyrin IX following exposure to 5-aminolaevulinic acid. *Photochem Photobiol* **62**: 887–895

Schick E, Kaufman R, Ruck A, Hainzl A and Boehncke WH (1995) Influence of activation and differentiation of cells on the effectiveness of photodynamic therapy. *Acta Dermatol Venerol* **75**: 276–279

Stratford IJ and Stephens MA (1989) The differential hypoxic cytotoxicity of bioreductive agents determined in vitro by the MTT assay. *Int J Radiat Oncol Biol Phys* **16**: 973–976

© Cancer Research Campaign 1997 

Van Steveninck J, Tijssen K, Boegheim JPJ, Van Der Zee J and Dubbleman TMAR (1986) Photodynamic generation of hydroxyl radicals by haematoporphyrin derivative and light. *Photochem Photobiol* **44**: 711–716

Vaupel P, Kallinowski F and Okunieff P (1989) Blood flow, oxygen and nutrient supply, and metabolic microenvironment of human tumours: a review. *Cancer Res* **49**: 6449–6465

Weishaupt KR, Gomer CJ and Dougherty TJ (1976) Identification of singlet oxygen as the cytotoxic agent in photo-inactivation of a murine tumour. *Cancer Res* **36**: 2326–2329

Yen A, Gigli I and Barrett KE (1990) Dual effects of protoporphyrin and long wave ultraviolet light on histamine release from rat peritoneal and cutaneous mast cells. *J Immunol* **144**: 4327–4332

© Cancer Research Campaign 1997